# Emergency Airway Management: A Systematic Review on the Effectiveness of Cognitive Aids in Improving Outcomes and Provider Performance

**DOI:** 10.3390/clinpract15010013

**Published:** 2025-01-06

**Authors:** Raisa Chowdhury, Ostap Orishchak, Marco A. Mascarella, Bshair Aldriweesh, Mohammed K. Alnoury, Guillaume Bousquet-Dion, Jeffrey Yeung, Lily Ha-Nam P. Nguyen

**Affiliations:** 1 Faculty of Medicine and Health Sciences, McGill University, Montreal, QC H3G 2M1, Canada; 2 Department of Otolaryngology-Head and Neck Surgery, McGill University, Montreal, QC H4A 3J1, Canada; 3 Department of Pediatric Surgery, McGill University, Montreal, QC H4A 3J1, Canada; 4 Department of Otolaryngology-Head & Neck Surgery, King Fahad Specialist Hospital Dammam, Dammam 32253, Saudi Arabia; 5 Department of Otolaryngology-Head & Neck Surgery, King Abdulaziz University, Jeddah 22254, Saudi Arabia; 6 Department of Anesthesia, McGill University, Montreal, QC H4A 3J1, Canada; 7 Institute of Health Science Education, McGill University, Montreal, QC H3G 2M1, Canada

**Keywords:** airway, cognitive aid, decision support tools, emergency, surgical airway

## Abstract

**Background/Objectives:** Emergency airway management is a critical skill for healthcare professionals, particularly in life-threatening situations like “cannot intubate, cannot oxygenate” (CICO) scenarios. Errors and delays in airway management can lead to adverse outcomes, including hypoxia and death. Cognitive aids, such as checklists and algorithms, have been proposed as tools to improve decision-making, procedural competency, and non-technical skills in these high-stakes environments. This systematic review aims to evaluate the effectiveness of cognitive aids in enhancing emergency airway management skills among health professionals and trainees. **Methods:** A systematic search of MEDLINE, Embase, CINAHL, Cochrane Library, Scopus, Web of Science, and ClinicalTrials.gov was conducted from February to March 2024. Studies examining the use of cognitive aids, such as the Vortex method, the ASA difficult airway algorithm, and visual airway aids, in emergency airway scenarios were included. Outcomes assessed included decision-making speed, procedural success rates, and non-technical skills. Data were extracted using standardized protocols, and the quality of included studies was appraised. **Results:** Five studies met inclusion criteria, encompassing randomized controlled trials, controlled studies, and mixed-methods research. Cognitive aids improved decision-making times (reduced by 44.6 s), increased procedural success rates, and enhanced non-technical skills such as teamwork and crisis management. Participants reported reduced anxiety and improved confidence levels (self-efficacy scores increased by 1.9 points). The Vortex method and visual cognitive aids demonstrated particular effectiveness in simulated scenarios. **Conclusions:** Cognitive aids significantly enhance emergency airway management skills, improving performance, reducing errors, and increasing provider confidence. Integrating cognitive aids into training programs has the potential to improve patient safety and outcomes. Further research is needed to validate these findings in clinical settings and optimize cognitive aid design and implementation.

## 1. Introduction

Healthcare providers, ranging from trainees to specialists, must efficiently manage acute airway emergencies [1]. These situations require high efficiency, a systematic approach, optimal crisis resource management, and multi-disciplinary collaboration. This is particularly true for ’cannot intubate, cannot oxygenate’ (CICO) scenarios, which, though rare, are encountered by anesthesiologists and intensivists on average 2.6 times throughout their careers [2]. The incidence of CICO is approximately 1:10,000–1:50,000 [3] of interventions under general anesthesia and such scenarios are even more commonly seen outside the operating room, with cricothyroidotomy occurring in about 0.2 to 1.2% of all tracheal intubations. CICO events carry a high risk of brain injury or death (25%) and impose significant emotional and cognitive burdens on providers [4,5,6].

CICO situations occur when conventional methods to oxygenate the patient, including facemask ventilation, insertion of a supraglottic airway device (SAD), and tracheal intubation, fail [7]. These failures can result in mortality due to brain hypoxia if a surgical airway is not promptly established [8]. The management of airway emergencies becomes more challenging and potentially severe when specialists such as otolaryngologists, trauma surgeons, or anesthetists, who are trained to perform critical interventions like cricothyrotomies, are hesitant or do not recognize the indication to perform such an intervention [9]. Additionally, the success rate of interventions is often lower due to infrequent training and the limited experience of healthcare providers [10]. Implementing cognitive aids has been proposed to address these challenges and effectively manage CICO situations. The Vortex approach is a cognitive aid designed to streamline airway management during critical events. It focuses on the three key lifelines of oxygenation—face mask ventilation, SAD insertion, and endotracheal intubation—prompting the clinician to cycle through those rapidly before moving to an emergency front-of-neck airway (FONA) if oxygenation remains unsuccessful. This method is intended to obviate delays and indecision through the simplification of decision-making during high-stress “cannot intubate, cannot oxygenate” situations [11,12]. The Difficult Airway Society guidelines reinforce this concept and position FONA as the fourth and last step, Plan D, in their airway management algorithms. The DAS promotes a scalpel–bougie cricothyroidotomy as the most reliable and universally applicable method for FONA because it is associated with higher success rates compared to other needle-based approaches [11,13]. FONA has classically been described as securing the airway via the anterior neck, most commonly via a cricothyroid membrane puncture or surgical cricothyroidotomy [11]. It is a life-saving intervention for the restoration of oxygenation and prevention of hypoxic injury when all other methods have failed [12].

Cognitive aids are increasingly used to enhance work performance and reduce errors [14]. These tools guide users in efficiently completing tasks while minimizing human errors or omissions [15]. They support cognitive processes such as attention, perception, and decision-making [16], and are utilized during task performance, unlike standard operating procedures [17]. Cognitive aids come in various forms, including algorithms, flowcharts, checklists, mnemonics, digital booklets, applications, and audiovisual prompts [14]. They must follow established guidelines, be tailored to specific situations, be recognizable to staff, and lead to an organized task completion [14].

In healthcare, cognitive aids are powerful tools for managing patients, especially during acute crises when time is critical and cognitive resources are limited. Examples include operating room emergency manuals such as the Stanford Emergency Manual, crisis checklists, the neuroanesthesia crisis manual, and the WHO Surgical Safety Checklist [17,18,19,20,21,22]. The value of cognitive aids in emergency airway management remains underexplored [23]. Efficient airway management is crucial for preserving oxygenation, ventilation, and hemodynamic stability during life-threatening emergencies [5]. However, the high-risk nature of these procedures can lead to practice variations and errors [24]. The literature does not conclusively determine the most effective cognitive aid for this field, and there are conflicting findings on their utility [25].

This systematic review evaluates the use of cognitive aids on the effectiveness, safety, and quality of emergency airway management, with a specific focus on ’cannot intubate, cannot oxygenate’ (CICO) scenarios. We assess their influence on procedural success rates, including first-pass and total success rates, and their potential to mitigate risks like hypoxia, hypotension, esophageal intubation, and airway trauma. Additionally, this review explores healthcare providers’ perceptions of cognitive aids, specifically in managing CICO situations. We also examine the role of visual or cognitive aids, such as cheat sheets, in enhancing procedural competency, reducing time to successful intervention, and decreasing error and failure rates during simulated surgical airway procedures. By investigating how medical professionals, residents, and trainees utilize these aids, we align our terminology and findings with the precise practices and expectations of anesthesiologists and other airway management experts.

## 2. Materials and Methods

### 2.1. Study Protocol

The systematic review was prepared in accordance with the Preferred Reporting Items for Systematic Reviews and Meta-Analyses (PRISMA) criteria. This protocol was registered with PROSPERO (CRD42024509860). The PICOT framework used in this systematic review focuses on healthcare providers (P) involved in managing critical airway emergencies, particularly CICO scenarios. The intervention (I) under evaluation is using cognitive aids, such as checklists, algorithms, and visual aids, compared to standard training or practice without such aids (C). The outcomes (O) measured include procedural success rates, the reduction of risks like hypoxia and hypotension, improved competency, and reduced error rates. The timeframe (T) primarily covers the immediate period during critical interventions, focusing on the minutes required to complete airway management procedures.

### 2.2. Study Selection

A comprehensive search strategy, developed with the assistance of a medical librarian, aimed to identify pertinent articles using seven electronic databases, from inception until 21 February 2024. The databases searched included Web of Science, MEDLINE (Pub-Med), Embase, CINAHL, Cochrane Library, Scopus, and ClinicalTrials.gov. As an example, the following search strategy was used to identify relevant studies in MEDLINE (Pub-Med): (“cognitive aid”[MeSH Terms] OR “cognitive aid” OR “checklist”[MeSH Terms] OR “checklist” OR “algorithm”[MeSH Terms] OR “algorithm” OR “decision support tool” OR “decision support system”[MeSH Terms] OR “flow chart” OR “protocol, clinical”[MeSH Terms]) AND (“emergency airway” OR “difficult airway” OR “intubation”[MeSH Terms]). The detailed search strategy can be found in supplemental Table S1 to evaluate the MeSH and keywords. This review focused on studies published in English and pertaining to human research. The term ’cognitive aids’ was specifically defined as tools that assist users in performing tasks or series of tasks during emergency airway management.

### 2.3. Inclusion/Exclusion Criteria

The inclusion and exclusion criteria (Table 1) were structured using the PICO framework to ensure clarity and focus on the research question. The Population (P) included studies focusing on adult airway management in simulated surgical settings, specifically utilizing adult mannequins. Pediatric airway management was excluded due to the lack of relevant studies meeting the inclusion criteria. The Intervention (I) encompassed the use of cognitive aids, such as cheat sheets or visual airway cognitive aids, to support airway management. The Comparator (C) was regular training without cognitive assistance or non-specific teaching strategies, and studies with more than one comparator or using unrelated interventions were excluded. The primary Outcomes (Os) included procedural competency, time to completion, procedure error rates, and surgical airway achievement rates in simulated scenarios. Secondary outcomes considered were the time taken for the first intervention, the number of attempts required, errors made, and the risk of harm. The inclusion criteria comprised quasi-experimental research, observational studies with control groups, controlled clinical trials (CCTs), and randomized controlled trials (RCTs). Studies such as reviews, editorials, case reports, case series, and uncontrolled observational studies were excluded.

### 2.4. Data Extraction and Synthesis

Two independent reviewers conducted data extraction using a standardized form (R.C. and O.O.). Extracted data included study characteristics, participant details, descriptions of the intervention and comparator, outcome data, and simulated scenario settings. Data were coded and classified based on important variables. The risk of bias in the included studies was evaluated using the Cochrane Risk of Bias Tool for RCTs and the ROBINS-I Tool for non-randomized studies [26,27]. Each study was evaluated on several domains, including random sequence generation, allocation concealment, the blinding of participants and personnel, the blinding of outcome assessment, incomplete outcome data, selective reporting, other biases, confounding, the selection of participants, the classification of interventions, deviations from intended interventions, missing data, the measurement of outcomes, and the selection of reported results. The risk of bias in each domain was categorized as “Low,” “Moderate,” “Unclear,” or “Not Assessed” based on predefined criteria. Two separate reviewers assessed the risk of bias in each study, and any differences were discussed to reach a consensus. If consensus was not reached, a third reviewer (L.N.) was consulted. We meticulously reviewed each study to ensure a comprehensive evaluation, with particular attention to domains critical to the integrity of the study outcomes. The data are extracted directly from the included studies, including means, standard deviations (SDs), and confidence intervals for key outcomes.

A narrative synthesis of the key findings from the included research was compiled to evaluate the outcomes of cognitive aids in airway management. This review focuses on summarizing study-level findings without conducting sensitivity or subgroup analyses.

## 3. Results

**Figure 1:**
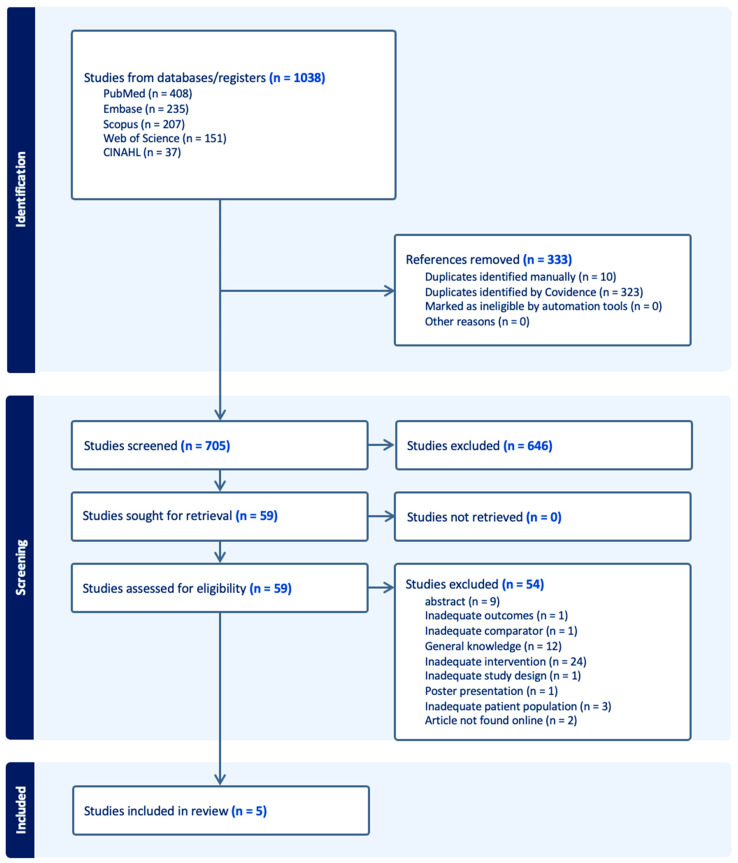
PRISMA flow diagram for study screening.

A total of 1038 studies were identified through database searches, including PubMed (*n* = 408), Embase (*n* = 235), Scopus (*n* = 207), Web of Science (*n* = 151), and CINAHL (*n* = 37). After removing 333 duplicates, 705 studies were screened based on title and abstract. Of these, 646 studies were excluded during the title and abstract screening phase for the following reasons: outcomes unrelated to procedural competency, error rates, or surgical airway success; comparators that did not isolate the effect of cognitive aids; or interventions not involving cognitive or visual aids. Subsequently, 59 studies were selected for full-text review, of which 54 were excluded for reasons such as irrelevance to the research question, use of study designs that did not meet the inclusion criteria (e.g., reviews, editorials, or case series), or patient populations that did not align with the scope of this review (e.g., pediatric studies). Five studies [6,10,28,29,30] were included in the final systematic review (Figure 1).

The included studies examined the impact of cognitive aids on emergency airway management in simulated settings. The findings revealed that cognitive aids, such as the American Society of Anesthesiologists (ASA) algorithm, the Vortex method, and visual airway cognitive aids, significantly improved healthcare professionals’ decision-making, procedural competency, and non-technical skills. These improvements were noted particularly in critical situations such as ’cannot intubate, cannot oxygenate’ (CICO) scenarios, where timely and accurate decisions are crucial for patient outcomes. These major findings of all included studies can be found in Table 2. A detailed summary of the outcomes measured, results, and statistical significance for each included study is presented in Table 3. This table provides a comprehensive comparison of procedural success, decision-making improvements, and other relevant outcomes observed in studies using cognitive aids for emergency airway management.

### 3.1. Competence

Two studies highlighted the effectiveness of cognitive aids compared with traditional training methods. The first study demonstrated improved surgical performance in anesthetic trainees using the Vortex technique alongside DAS recommendations, where the time to perform FONA was reduced from fifty-six point eight seconds to forty-four seconds, and self-efficacy increased from fifty percent to eighty-seven point five percent (*p* < 0.001) across three simulation sessions. Self-efficacy was measured using a validated scale that assesses confidence in performing specific tasks under simulated conditions. Another study evaluated anesthetists who received formal emergency front-of-neck access (eFONA) training under DAS guidelines, showing a considerable increase in their ability to recognize CICO scenarios and perform eFONA in less than 10 min. The post-training success rate improved by 12.1% compared to the pre-training rate, indicating significant improvement.

### 3.2. Time to Decision-Making

Two studies explicitly evaluated time to decision-making. Zasso et al. (2021) [30] found that anesthetists who received formal FONA training showed improved recognition and execution of FONA operations in simulated airway crises. Visual airway cognitive aids were associated with higher checklist scores and faster decision times for FONA, with a mean difference in decision durations of −8.5 s (95% CI [−13.1, −3.9], *p* < 0.001). Berwick et al. (2019) [28] demonstrated a reduction in the total time required to perform a surgical cricothyrotomy, with the median time decreasing from 225 s to 151.5 s (*p* = 0.002), mainly due to a decrease in decision-making time by an average of 25.5 s (*p* = 0.027).

### 3.3. Technical and Non-Technical Skills

One study concluded that cognitive aid use improved non-technical skills but did not significantly enhance technical skills during simulated CICO emergencies. Compared to the control group, the cognitive aid group’s clinicians demonstrated a notably higher percentage of effective oxygenation within less than 10 min (odds ratio: 3.18, 95% CI [1.12, 9.00], *p* = 0.030). The cognitive aid group also showed better Anesthetists’ Non-Technical Skills (ANTSs) scores, indicating enhanced non-technical abilities.

### 3.4. Load and Anxiety

The Vortex method proved simpler and more convenient in two different studies, with one study showing a reduced load compared to another algorithm. This study compared the ASA difficult airway algorithm and the Vortex method in research involving medical students, revealing significant differences. Students trained using the Vortex technique outperformed those trained with the ASA algorithm in airway management scores and completeness, with a mean difference of 1.24 (95% CI [0.52, 1.96], *p* = 0.002). Post-simulation, both groups experienced increased anxiety, with the ASA group showing higher NASA-TLX ratings, though not significantly (mean difference = 5.72, 95% CI [−0.89, 12.33], *p* = 0.091).

### 3.5. Risk of Bias Assessment

**Figure 2:**
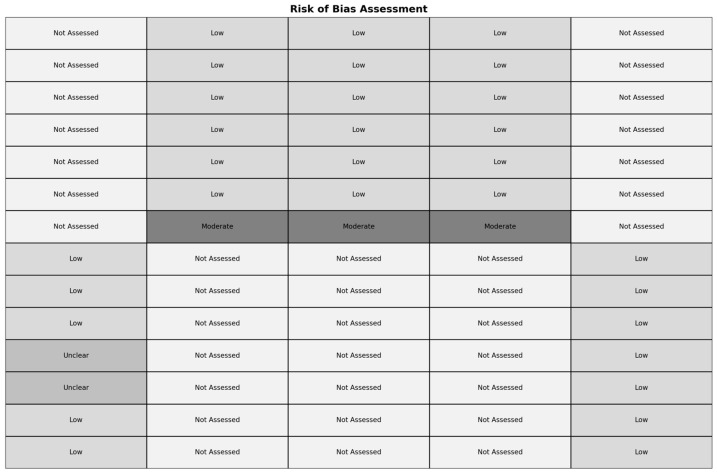
Risk of Bias Assessment Across Included Studies. The figure presents the risk of bias evaluation for various domains across the studies included in the analysis.

The risk of bias assessment revealed variability across the included studies (Figure 2). For instance, Ambardekar et al. (2019) [6] demonstrated a low risk of bias in most assessed domains but had “Unclear” risks associated with the blinding of participants and personnel, as well as the blinding of outcome assessment. In contrast, Berwick et al. (2019) [28] and Marshall et al. (2014) [10] were not assessed for many domains but presented a moderate risk of confounding. O’Sullivan et al. (2023) [29] and Zasso et al. (2021) [30] showed low risks across the evaluated domains, although certain key areas, such as confounding and the selection of participants, were not assessed. These findings highlight the need for the cautious interpretation of results, particularly in studies with identified risks, to mitigate potential bias in the analysis and conclusions.

## 4. Discussion

This review is the first to focus on the use of cognitive aids in emergency airway management. Despite some limitations, including the small number and heterogeneity of the studies, the findings indicate the significant benefits of cognitive aids in improving essential performance metrics for efficient emergency airway care [6,10,30]. While not every study offered statistical findings, qualitative results provided valuable insights into the perceived effects of cognitive aid deployment on trainees’ confidence and skill acquisition. O’Sullivan et al. (2023) [29] similarly found increased confidence and success rates with formal eFONA training in an obstetric context, despite the absence of statistical data.

Cognitive aids have been shown to enhance competency, non-technical skills, and decision-making time across different emergency airway management scenarios, including both CICO situations and other urgent intubation needs. These aids enable healthcare professionals to see the bigger picture, follow algorithms, and avoid fixation, thereby moving forward when initial plans to secure the airway have failed [4]. Given the critical importance of rapid airway management in the resuscitation of patients requiring urgent care, the observed decrease in the time to successful intubation with cognitive aid application is very notable. Our results are in line with other research showing a link between extended efforts at intubation and unfavorable outcomes, such as hypoxemia and cardiac arrest [6,31]. Additionally, several other performance indications were significantly improved, including first-pass success rates, the duration to successful intubation, and the frequency of airway-related problems. These outcomes are reinforced by other studies [32,33] that demonstrated the usefulness of cognitive aids in enhancing procedural performance across various medical specialties.

Despite the numerous amounts of cognitive aids utilized within healthcare, very few had been utilized for the purpose of emergency airway management, including the Vortex method, the ASA algorithm, and visual airway cognitive aids. While each aid showed benefits, the review did not conclusively establish one as superior to the others with respect to best-practice guidelines.

There is a lack of randomized controlled trials (RCTs) comparing different cognitive aid tools designed to assist healthcare professionals during CICO events, making it difficult to determine the superiority of one tool over another. Moreover, if a decision is made to implement a particular cognitive aid tool, the local team should ensure that the proposed algorithm aligns with standards of best practice and available resources.

On the matter of beneficiaries of cognitive aids, the review found that both trainees and experienced anesthetists gained from the use of cognitive aids. Trainees showed increased performance and self-efficacy indicating that these tools have the potential to standardize and improve the delivery of care in emergency situations [34,35]. Similarly, experienced anesthetists demonstrated an improved ability to recognize and carry out procedures. This finding is pertinent, as skilled healthcare providers, be they technicians, surgeons, or specialists, should not eschew these aids for fear of being viewed as inexperienced or take risks when deciding on critical steps in managing patients.

The studies also did not explicitly address potential barriers to using cognitive aids in acute settings or discuss alternative approaches, so we cannot conclude whether these same cognitive aids used in the studies would work seamlessly in a real-world setting. Every tool requires knowledge, skills, and constant practice to effectively utilize it in critical situations. Thus, repeated simulation trainings should be used alongside with implementation of cognitive aid tool to improve effectiveness and help healthcare professionals retain skills over time [36]. Depending upon the robustness of the healthcare service or facility within a particular setting, there could be numerous obstacles and hinderances within the clinical environment, be it the emergency department, the intensive care unit, or even the operating theater within a hospital. Which is why the true strength of a cognitive aid can accurately be assessed only after it has been implemented in various clinical settings (e.g., hospitals amidst war zones, overcrowded medical centers, hospitals with poor financing, etc.).

Finally, our data demonstrate how cognitive assistance might reduce unfavorable outcomes and enhance overall procedural quality, as seen by the lower frequency of airway-related problems. This indicates the significance of incorporating evidence-based therapies, including cognitive aids, into clinical practice to enhance patient outcomes and is in line with the objectives of patient-centered care [37,38]. Further studies assessing the use of cognitive aids in real-world scenarios are necessary to determine their reliability, particularly in emergency airway management. Universities should disseminate well-designed cognitive aids online, and it is crucial to address the misconception that using cognitive aids denotes a lack of experience; both trainees and experienced personnel can benefit from them.

## 5. Limitations

Although our research yielded encouraging results, it is important to consider a few limitations when interpreting the data. First, the generalizability of our findings may be limited due to the small number of included studies (*n* = 5). This reflects the lack of extensive research on cognitive aids in emergency airway management, particularly in “cannot intubate, cannot oxygenate” (CICO) scenarios. While our stringent inclusion criteria were designed to ensure methodological quality, they also contributed to a limited sample size, which reduces external validity. Further research with broader inclusion criteria or larger, multi-center studies is needed to validate these findings across diverse clinical settings. Second, the heterogeneity of the included studies regarding research design, participant characteristics, and outcome measures introduces a degree of uncertainty in the pooled conclusions. Subgroup analyses were not conducted due to insufficient data and variability in study methodologies, which made statistical comparisons unfeasible. Future research should aim to address this limitation by exploring the differential impacts of cognitive aids across participant characteristics, intervention types, and clinical contexts. Third, the majority of the included studies were conducted in simulated, controlled settings, which may not fully replicate the complexities, resource constraints, and stress levels experienced during real-world clinical emergencies. While simulations provide valuable insights into procedural competency and decision-making, they fail to account for factors such as heightened stress, patient variability, and time pressures that influence outcomes in actual clinical practice [39,40]. This limitation underscores the need for further studies conducted in real-world settings to evaluate the practical feasibility and effectiveness of cognitive aids under realistic conditions. Lastly, there is still uncertainty regarding the long-term effects of cognitive aid implementation on patient-centered outcomes, such as morbidity, mortality, and resource utilization. To address this, additional prospective, multi-center studies with sufficient follow-up periods are required to assess the sustained impact of cognitive aids in emergency airway management [36,41]. Despite these limitations, our findings provide valuable insights into the potential role of cognitive aids in improving decision-making, procedural success, and provider confidence during airway emergencies.

### Summary of Cognitive Aids in Emergency Airway Management

We have included a summary of important cognitive aids (Table 4) in emergency airway management to supplement the findings of this systematic review and improve its applicability in clinical practice and education. For healthcare professionals, educators, and trainers, this synopsis is a useful resource that offers comprehensive understanding of the instruments that can facilitate efficient airway management in emergency situations.

## 6. Conclusions

The findings of this systematic review highlight the potential for cognitive aids to enhance emergency airway management outcomes significantly. The robust data suggest that incorporating cognitive aids such as the Vortex method, the ASA difficult airway algorithm, and visual airway cognitive aids into clinical practice and training programs can improve patient outcomes, reduce errors, and enhance provider performance in emergency airway settings. This evidence supports the broader integration of cognitive aids in clinical protocols to optimize the efficiency and effectiveness of emergency airway management.

## Figures and Tables

**Table 1:** The inclusion and exclusion criteria of the systematic review.

Inclusion Criteria	Exclusion Criteria
Healthcare Trainees: physicians, clinicians, medical residents and students/trainees participating in emergency airway management training.	Non-Relevant Outcomes: studies not reporting outcomes related to procedural competency, time to completion, error rates, or success rates in achieving a surgical airway.
Surgical Airway Scenarios: studies focusing on emergent surgical airway management.	Non-English Studies: the exclusion of studies published in languages other than English due to language proficiency constraints.
Cognitive Aid Implementation: studies involving the use of cheat sheets as cognitive or visual aids.	Studies with Insufficient Data: studies with insufficient data were excluded, specifically those lacking clear reporting on procedural success rates, error rates, or any outcomes directly measuring the effectiveness of cognitive aids in emergency airway management. Non-Peer-Reviewed Literature: the exclusion of non-peer-reviewed literature, conference abstracts, and unpublished studies.
Outcome Measurements: studies reporting outcomes related to procedural competency, time to completion, error rates during the procedure, and success rates in achieving a surgical airway.	Studies Predominantly Focusing on Non-Relevant Interventions: the exclusion of studies where the primary focus is on interventions unrelated to cognitive aids.

**Table 2:** Major findings of the five studies for the systematic review.

Study	Year	Study Design	Location	Objective	Participants	Intervention	Outcome	Aids Used	Sample Size	Conclusion
Ambardekar et al. [6]	2019	Prospective randomized controlled trial	USA	To compare the Vortex approach and the ASA difficult airway algorithm regarding learners’ decision-making skills during difficult airway management.	Third- and fourth-year medical students	Training with either the Vortex approach or the ASA difficult airway algorithm	Students in the Vortex group had higher airway management scores and completeness compared to the ASA group. Anxiety scores increased in both groups after simulation, with higher NASA-TLX scores in the ASA group.	Vortex approach and ASA algorithm	Total of 67 participants (ASA group: 33, Vortex group: 34)	Training in the simpler Vortex approach improved decision-making skills during difficult airway management.
Berwick et al. [28]	2019	Prospective mixed-methods pilot study	England	To develop and evaluate a training package combining surgical teaching with instruction on DAS 2015 guidelines and the Vortex cognitive aid for emergency surgical cricothyroidotomy.	Anesthetic specialist trainees (years 5–7)	Training package incorporating DAS guidelines and Vortex cognitive aid	Improved consistency of trainee performance, increased self-efficacy for managing CICO emergencies, and adoption of the Vortex approach in managing airway crises.	Vortex cognitive aid	Total of 10 participants	The training package improved trainee performance and self-efficacy in managing CICO emergencies.
Marshall et al. [10]	2014	Prospective study	Australia	To determine the effects of displaying a cognitive aid during a simulated CICO crisis on critical care specialists and anesthetists’ management.	Experienced critical care clinicians	Display of a cognitive aid during simulation	Higher proportion of clinicians in the cognitive aid group could oxygenate within 3 min. ANTS scores were higher when a cognitive aid was supplied.	Cognitive aid–CICO linear flow chart	Total of 64 participants (control: 38, intervention: 26)	Use of a cognitive aid improved non-technical skills and facilitated quicker oxygenation in simulated CICO crises.
O’Sullivan et al. [29]	2023	Controlled study	Ireland	To assess the impact of introducing formal eFONA training in an obstetric hospital’s perioperative medicine department.	Anesthetists	eFONA training	Improved success rates and confidence in identifying and performing eFONA.	None	Total of 17 participants	Formal eFONA training improved participants’ success rates and confidence in managing eFONA scenarios.
Zasso et al. [30]	2021	Randomized controlled study	Canada	To investigate the effects of a visual airway cognitive aid on decision-making in simulated airway emergency scenarios.	Resident teams, nurses, respiratory therapists	Use of a visual airway cognitive aid	Shorter decision time for front-of-neck access and higher checklist scores when the cognitive aid was used.	2013 American Society of Anesthesiologists Difficult Airway Algorithm	Total of 40 teams (control: 20, intervention: 20)	Prior exposure to a visual airway cognitive aid improved decision-making in simulated airway emergencies.

**Table 3:** Outcome Measures of Cognitive Aid Interventions Across Included Studies.

Study	Outcome Measured	Results	Statistical Significance	Observations
Ambardekar et al. [6]	Decision-making score	Vortex: Median 4.0 (IQR 4.0–5.0), ASA: Median 4.0 (IQR 3.0–4.0)	*p* = 0.0003	Vortex group had better decision-making skills.
	Completeness	Vortex: 94.1%, ASA: 63.6%	*p* = 0.003	Completeness was significantly higher in the Vortex group.
	Cognitive task load (NASA-TLX)	Mental demand: ASA: 61.4 (SD 14.4), Vortex: 51.0 (SD 22.7)	*p* = 0.030	Vortex reduced cognitive load for mental demand.
	Anxiety (STAI-Y)	Post-simulation: ASA: 13.4 (SD 3.4), Vortex: 12.3 (SD 3.9)	*p* = 0.138	Anxiety increased in both groups; no significant difference between groups.
Berwick et al. [28]	Deliberation time to initiate surgical cricothyroidotomy	Decreased from 101.5 s (IQR 92.25–177.5) in simulation A to 76.0 s (IQR 63.25–92.0) in simulation C	*p* = 0.027	Training reduced deliberation time, reflecting improved decision-making skills.
	Total time to perform SCT	Decreased from 225.0 s (IQR 163.8–244.0) in simulation A to 151.5 s (IQR 129.3–190.0) in simulation C	*p* = 0.002	The reduction in total time was attributed to faster deliberation rather than surgical execution.
	Self-efficacy score	Increased from 50% (median) in simulation A to 87.5% in simulation C	*p* < 0.001	Significant improvement in confidence six months after training.
	Concordance with DAS guidelines	Improved from 60% (simulation A) to 100% (simulation C)	Not significant (*p* = 0.134)	Progress towards adherence with vertical incision and guideline-based execution.
Marshall et al. [10]	Anesthetists’ Non-Technical Skills (ANTSs) total score	Control: 10.4 (SD 3.1); Intervention: 13.2 (SD 2.4)	*p* < 0.001	Cognitive aids significantly improved team behaviors in all ANTS categories.
	Time to provide oxygenation	Control: 183.8 s (SD 65.0); Intervention: 165.4 s (SD 64.4)	*p* = 0.27	No statistically significant difference in time to oxygenation.
	Oxygenation provided within 3 min	Control: 55.3%; Intervention: 76.9%	*p* = 0.076	A trend toward faster oxygenation in the cognitive aid group.
	Team management score	Control: 2.5 (SD 0.8); Intervention: 3.2 (SD 0.8)	*p* = 0.002	Improved ability to organize and lead the team in crisis scenarios.
	Situation awareness score	Control: 2.6 (SD 0.8); Intervention: 3.5 (SD 0.6)	*p* < 0.001	Marked improvement in awareness of team needs and scenario dynamics.
	Evidence of conflict	Control: 5 incidents; Intervention: 0 incidents	*p* = 0.066	Conflict incidents reduced to zero in the cognitive aid group.
O’Sullivan et al. [29]	Time to successful lung inflation	Pre-intervention: 123.6 s (SD 69.4); Post-intervention: 80.8 s (SD 46.3)	*p* = 0.0192	Significant reduction in procedural time after eFONA training intervention.
	Success rate for lung inflation within 240 s	Pre-intervention: 82%; Post-intervention: 94.1%	Not reported	Improved success rate post-training.
	Ease of procedure (VAS score)	Pre-intervention: 4.13 (SD 2); Post-intervention: 7.47 (SD 1.5)	*p* < 0.0001	Significant improvement in perceived ease of performing the procedure.
	Confidence in identifying eFONA need	Pre-intervention: 7/17 “very unconfident”; Post-intervention: 16/17 reported improved confidence	Not reported	Training improved participants’ confidence in identifying the need for eFONA.
	Skill retention at 3 months	Time to lung inflation: Improvement of 12 s (mean) compared to first attempt (80.18 s)	*p* = 0.68	No statistically significant improvement in skill retention over 3 months.
Zasso et al. [30]	Decision-making time to perform FONA	Intervention: 80.9 ± 54.5 s; Control: 122.2 ± 55.7 s; Difference: −41.2 s (95% CI: −76.5 to −6.0)	*p* = 0.023	Intervention group made decisions faster than the control group.
	Cognitive aid usage rate	Intervention: 63.0%; Control: 28.1%	*p* < 0.001	Intervention group relied more on cognitive aid compared to the control group.

**Table 4:** Summary of Cognitive Aids for Enhancing Emergency Airway Management.

Cognitive Aid	Description	Application	Key Findings	Comments
**Vortex Method**	A structured approach designed to guide decision-making during difficult airway scenarios.	It is commonly used in simulated airway emergencies to streamline decision-making processes.	Improved decision-making time and increased success rates in simulated environments.	Recommended for inclusion in emergency airway protocols due to its simplicity and effectiveness.
**ASA Difficult Airway Algorithm**	A detailed algorithm by the American Society of Anesthesiologists for managing difficult airways.	Widely applied in both clinical and simulated settings to standardize airway management procedures.	Enhanced consistency in performance and reduced anxiety among healthcare providers.	Effective for both training purposes and real-world application, especially in high-stress situations.
**WHO Surgical Safety Checklist**	A checklist designed to improve safety and reduce errors during surgical procedures, including airway management.	Used to ensure all critical steps are completed correctly during airway management.	Significantly reduced the incidence of complications and improved overall safety.	A key tool for integrating cognitive aids into broader patient safety initiatives.
**Visual Airway Cognitive Aid**	A visual aid designed to prompt key steps and decisions during airway emergencies.	Applied in simulated environments to assist with decision-making during critical airway scenarios.	Shortened decision-making time and improved checklist adherence.	It is particularly useful in fast-paced scenarios where quick decisions are crucial.

## Data Availability

Data extracted from literature material can be made available upon reasonable request to the corresponding author.

## Supplementary Materials

The following supporting information can be downloaded at: https://www.mdpi.com/article/10.3390/clinpract15010013/s1, Table S1: Search Strategy.
