# Supplementary material for: Emergency Airway Management: A Systematic Review on the Effectiveness of Cognitive Aids in Improving Outcomes and Provider Performance
Source: Clin Pract. 2025 Jan 6;15(1):13. doi: 10.3390/clinpract15010013 (PMC11764273; doi:10.3390/clinpract15010013)
Supplement: Supplementary file 1 [file clinpract-15-00013-s001.zip › Supplementary Table S1.pdf]

Supplementary Table S1: Search Strategy

Database	Search Terms (MeSH and Keywords)	Boolean Logic
**MEDLINE (PubMed)**	("cognitive aid"[MeSH Terms] OR "cognitive aid" OR "checklist"[MeSH Terms] OR "checklist" OR "algorithm"[MeSH Terms] OR "algorithm" OR "decision support tool" OR "decision support system"[MeSH Terms] OR "flow chart" OR "protocol, clinical"[MeSH Terms]) AND ("emergency airway" OR "difficult airway" OR "intubation"[MeSH Terms])	AND "humans"[MeSH Terms] AND "English"[Language]
**Web of Science**	("cognitive aid" OR "checklist" OR "algorithm" OR "decision support tool" OR "flow chart" OR "protocol, clinical") AND ("emergency airway" OR "difficult airway" OR "intubation") AND ("cricothyrotomy" OR "front of neck access" OR "surgical airway")	AND "humans" AND "English"
**Embase**	('cognitive aid'/exp OR 'cognitive aid' OR 'checklist'/exp OR 'checklist' OR 'algorithm'/exp OR 'algorithm' OR 'decision support tool' OR 'flow chart' OR 'clinical protocol'/exp) AND ('emergency airway' OR 'difficult airway' OR 'resuscitation' OR 'intubation')	AND 'human'/exp AND 'English'/exp
**CINAHL**	("cognitive aid" OR "checklist" OR "algorithm" OR "decision support tool" OR "flow chart" OR "clinical protocol") AND ("emergency airway" OR "difficult airway" OR "resuscitation" OR "intubation" OR "cricothyrotomy" OR "front of neck access" OR "surgical airway")	AND "humans" AND "English"
**Cochrane Library**	("cognitive aid" OR "checklist" OR "algorithm" OR "decision support tool" OR "flow chart" OR "clinical protocol") AND ("emergency airway" OR "difficult airway" OR "resuscitation" OR "intubation" OR "cricothyrotomy" OR "front of neck access" OR "surgical airway")	AND "humans" AND "English"
**Scopus**	TITLE-ABS-KEY("cognitive aid" OR "checklist" OR "algorithm" OR "decision support tool" OR "flow chart" OR "clinical protocol") AND TITLE-ABS-KEY("emergency airway" OR "difficult airway" OR "resuscitation" OR "intubation"	AND "humans" AND "English"

	OR "cricothyrotomy" OR "front of neck access" OR "surgical airway")	
**ClinicalTrials.gov**	("cognitive aid" OR "checklist" OR "algorithm" OR "decision support tool" OR "flow chart" OR "clinical protocol") AND ("emergency airway" OR "difficult airway" OR "resuscitation" OR "intubation" OR "cricothyrotomy" OR "front of neck access" OR "surgical airway")	AND "humans" AND "English"
